# Interferon-Type-I Response and Autophagy Independently Regulate Radiation-Induced HLA-Class-I Molecule Expression in Lung Cancer

**DOI:** 10.3390/cimb48010028

**Published:** 2025-12-25

**Authors:** Erasmia T. Xanthopoulou, Ioannis Lamprou, Ioannis M. Koukourakis, Achilleas G. Mitrakas, Georgios D. Michos, Anastasia Polyzoidou, Filippos G. Antoniadis, Alexandra Giatromanolaki, Michael I. Koukourakis

**Affiliations:** 1 Department of Radiotherapy/Oncology, Democritus University of Thrace, University Hospital of Alexandroupolis, 68100 Alexandroupolis, Greecelambrou87bio@gmail.com (I.L.); ikoukourakis@med.uoa.gr (I.M.K.); amitrak@med.duth.gr (A.G.M.); geormichos@yahoo.gr (G.D.M.); anapol24801@gmail.com (A.P.); filipposandoniadis@gmail.com (F.G.A.); 2 Department of Pathology, Democritus University of Thrace, University Hospital of Alexandroupolis, 68100 Alexandroupolis, Greece

**Keywords:** radiotherapy, HLA, autophagy, IFN-type-I, lung cancer, JAK, TBK1

## Abstract

**Background/Objectives**: The enhancement of antitumor immune responses by radiotherapy (RT) is partially attributed to the activation of the IFN-type-I pathway. However, the loss of HLA-class-I molecules, which occurs in a large percentage of non-small-cell lung cancers (NSCLCs), may block the cytotoxic effect of T-cells and immunotherapy (IO). Moreover, autophagy is also involved in HLA downregulation. We investigated the complex interactions between RT, HLA molecules, autophagy, and IFN-type-I responses. **Methods**: The A549, H1299, and ATG7-deficient NSCLC cell lines, along with the modified shLC3A H1299 cell line, were used for in vitro experiments. The effect of RT (8 and 3 × 8 Gy) on Interferon beta (IFNβ), IFN-stimulated genes (ISGs), and HLA-class-I expression in combination with IFN-type-I-response inhibitors (Ruxolitinib, Tofacitinib, Amlexanox) targeting the JAK and TBK1 was studied with Flow cytometry and RT-PCR. **Results**: RT significantly induced HLA-class-I expression. A parallel upregulation of IFNβ and ISGs mRNA levels was also documented. Although the IFN-type-I-response inhibitors suppressed the RT-induced IFNβ and ISGs expression, their effect on HLA-class-I expression was minimal. Blockage of LC3A autophagy (shLC3A cell line) significantly upregulated HLA-class-I basal levels, and RT further enhanced HLA expression. IFN-type-I-response inhibitors blocked the RT-inductive effect in the shLC3A H1299, but had no effect in the ATG7-deficient H1650 cell line. **Conclusions**: The current study supports the theory that baseline autophagy, RT-induced autophagy blockage, and IFN-type-I response enhancement define the HLA-class-I levels in NSCLC cells. This complex interplay emerges as a promising target for the development of radio-vaccination strategies to enhance the efficacy of radio-immunotherapy.

## 1. Introduction

Lung cancer ranks among the leading causes of cancer-related deaths, with projections indicating that its incidence may double by the year 2050 [1]. Frequently, this type of cancer is identified at an advanced stage, and curability after chemotherapy and RT is unacceptably low [2]. Numerous targeting drugs, inhibitors of EGFR, ALK, RAS-MAPK, are available in clinical practice, prolonging survival in relatively small subgroups of patients [3]. A deeper understanding of lung cancer biology has also facilitated the advancement of immunotherapy (IO). Immune checkpoint inhibitors (ICIs) have demonstrated advantages in lung cancer treatment and are often used in conjunction with chemotherapy and RT to enhance their efficacy. RT is the basis for the treatment of locally advanced lung cancer, and anti-PD1 immunotherapy after chemo-RT has significantly improved the survival and even the curability of the disease [4].

Radiation-induced cell killing involves the formation of double-strand DNA breaks resulting from the interaction of free radicals produced by ionizing radiation with cellular molecules [5]. This very effect, among others, promotes interesting interactions between cancer cells and the immune response. Fragmented double-stranded DNA enters the cytoplasm, activating the cGAS-STING pathway, driving the transcription of various ISGs, thus triggering the IFN-Type I response [6,7]. Tumor-infiltrating antigen-presenting dendritic cells are activated, stimulating cytotoxic in situ or lymph-node-residing T-cells, enabling them to recognize and kill cancer cells [8]. Radiotherapy also indirectly activates DCs by promoting Flt3L release from damaged bone marrow cells and fibroblasts [9]. In a recent study, Kuncman et al. examined the kinetics of Flt-3L in the plasma of patients with rectal cancer undergoing neoadjuvant chemoradiotherapy, demonstrating a significant increase during radiotherapy [10]. The magnitude of this rise was inversely related to lymphopenia.

HLA-class-I are IFN-inducible genes [11], responsible for forming the HLA complex that presents foreign peptides produced by the intracellular degradation of abnormal proteins. This is essential for cytotoxic T-cells to recognize and destroy cancer cells. HLA-class-I expression is often lost during the immunoediting process; as shown in our recent study, this occurs in 65% of human non-small-cell lung cancers (NSCLCs) [12]. Cancer cell irradiation has been shown to induce HLA-class-I expression [13], which may be related to the radiation-induced IFN-type-I response.

Additional pathways might also contribute. As shown earlier, autophagy inhibitors induce HLA-class-I expression in lung cancer cells, and the disruption of autophagic flux caused by RT could also be a factor [14,15]. Lipidation of LC3, a key protein involved in autophagy, has been shown to be an essential step for the internalization and degradation of membrane HLA-class-I proteins [16]. In a recent study, Yamamoto et al. showed that autophagy plays a vital role in degrading HLA in pancreatic cancer [17].

Autophagy serves as an adaptive response to different stresses, such as starvation and oxygen deprivation [18]. Misfolded and damaged proteins and organelles are enclosed in double-membrane autophagosomes, which fuse with lysosomes to break down their contents into simple molecules like amino acids and fatty acids. These molecules are subsequently recycled for energy generation and membrane synthesis. The formation of autophagosomes starts with the development of a double lipid membrane that contains lipidated ATG microtubule-associated proteins (MAP1LC3, LC3s). The three recognized members of the LC3 family (LC3A, LC3B, and LC3C) show different kinetics and may perform different biological functions [19]. LC3A is often overexpressed in tumors, including lung cancer, and associated with poor prognosis [20]. LC3 can be identified as a proform of LC3, which is first synthesized as a full-length protein that is subsequently cleaved at the C-terminus by the enzyme Atg4B to generate a cytosolic form, LC3-I. For the autophagosome formation, LC3-I is converted into a lipidated (membrane-associated) form the LC3A-II [21]. The fusion of autophagosomes with lysosomes requires the presence of Lysosome-associated membrane proteins (LAMPs), which are essential for regulating the autophagy process [22]. An important molecule involved in loading proteins into autophagosomes for degradation is the sequestosome 1/p62 protein, which acts as an adaptor linking ubiquitinated cargo to LC3 proteins on the autophagosomal membrane [23]. p62 is subsequently broken down as autophagosomes are degraded. These markers collectively facilitate the study of autophagy status and the complex dynamics of autophagy flux, focusing on autophagosome formation and auto-lysosomal degradation.

Many studies in the literature indicate that RT influences autophagic flux, although this interaction is intricate. RT can lead to both increased and blocked autophagic flux, with the specific effect depending on cell type, genetic background, and radiation dose [24,25]. Larger doses may inhibit autophagy for several days, followed by a reactive increase in cells surviving the radiation damage [25]. In any case, the molecular mechanisms of RT’s interaction with autophagy remain unclear.

In this study, we examined the hypothesis that RT can increase the expression of HLA-class-I molecules through different pathways, involving the IFN-type-I response and autophagy inhibition. This can significantly influence the design of trials focused on boosting the activity of anti-PD1/PD-L1 immunotherapy through RT and its combinations with IFNs-type-I and autophagy inhibitors. Additionally, using RT and autophagy inhibitors to induce HLA-class I may reactivate ICI effectiveness in patients resistant to immunotherapy [26].

## 2. Materials and Methods

### 2.1. Cell Lines—Cell Culture Conditions and Irradiation

A549 and H1299 NSCLC cell lines, as well as the ATG7-deficient H1650 cell line, were cultured in DMEM Low Glucose (Dulbecco’s Modified Eagle Medium; Biosera, Cholet, France, enriched with 10% (*v*/*v*) 10% Fetal Bovine Serum (PAN-Biotech, Aidenbach, Germany) and 1% (*v*/*v*) 1% Penicillin/Streptomycin [Cat no: 15140-122; Gibco, Life Technologies, Carlsbad, CA, USA], at 37 °C in a humidified incubator with 5% CO_2_ (Heraeus, New York, NY, USA, 12L01I001)). The A549, H1299, and H1650 cell lines were obtained from ATCC (Manassas, VA, USA, ATCC^®^ CCL-185TM, ATCC^®^ CRL-5803TM, ATCC^®^ CRL-5883TM, respectively). Further information about these cell lines is available at https://www.atcc.org/Products/All/CCL-185.aspx?slp=1#generalinformation, accessed on 12 December 2025, http://www.atcc.org/Products/All/CRL-5803.aspx#characteristics, accessed on 12 December 2025, https://www.atcc.org/products/crl-5883, accessed on 12 December 2025. The authentication of the cell lines was performed by ATCC, and additional details regarding the characterization methods can be found at http://www.atcc.org/Products/Cells_and_Microorganisms/Testing_and_Characterization/STR_Profiling_Analysis.aspx, accessed on 12 December 2025. Cells were exposed to either a single dose of 8 Gy or three daily fractions of 8 Gy, using a 6-MV beam produced by an InfinityTM linear accelerator (Elekta, Stockholm, Sweden). The ATG7-deficient H1650 cell line, which has lost ATG7 expression due to a focal biallelic deletion within the ATG7 locus [16], was gifted to our Department by Professor Adrial L. Harris, Cancer Research, UK.

The analysis of the irradiated cell lines was conducted at 72 h in all experiments. This time point was selected based on prior experiments with autophagy blockers [14], where HLA induction was clearly evident at 72 h but not at 24 h. Furthermore, since experiments were based on one fraction and three daily fractions of 8 Gy, it is important to select the 72 h time point (24 h after the last fraction) for both regimens.

All experiments performed in this study were conducted in triplicate.

### 2.2. IFN-Type-I Pathway Inhibitors

Since RT induces an IFN-type-I response and the secretion of relevant IFNs, we designed experiments that block downstream molecular events of IFNβ using specific inhibitors to test how this blockage affects HLA-class-I expression. Tofacitinib, a Janus kinase (JAK) inhibitor, mainly influences intracellular signaling pathways associated with various cytokines and growth factors. By inhibiting JAK enzymes, especially JAK1 and JAK3, Tofacitinib decreases the signaling downstream of IFNβ and overall IFN-Type I responses. As a result, even in the presence of IFNβ, the cellular response may be weakened due to the incomplete activation of essential signaling pathways caused by JAK enzyme inhibition [27]. Ruxolitinib, another JAK inhibitor, mainly targets JAK1 and JAK2. Like tofacitinib, Ruxolitinib inhibits the JAK-STAT signaling pathway, which is crucial for the phosphorylation and activation of STAT proteins necessary for relaying signals from the IFNβ receptor. This inhibition leads to a diminished cellular response to IFNβ, potentially reducing its effectiveness in modulating the immune response and exerting antiviral effects [28]. Amlexanox is recognized as an inhibitor of TANK-binding kinase 1 (TBK1) and IκB kinase ε (IKKε) [29]. These kinases play a crucial role in regulating the IFN-type-I response, particularly in the production of IFNβ. Through the inhibition of TBK1 and IKKε, Amlexanox diminishes the phosphorylation of IRF3, a process essential for the transcription of the IFN-β gene. By using Amlexanox, we anticipated that we would be able to mitigate the IFN-type-I response more effectively and selectively, not only by limiting the paracrine loop signaling of IFNβ.

To evaluate their effect on IFNβ expression in the concept of lung cancer, we purchased the three inhibitors Tofacitinib [Cat no: 11598; Lot no: 0608331-42; CAYMAN CHEMICAL, USA], Ruxolitinib [Cat no: 11609; Lot no: 0568069-83; CAYMAN CHEMICAL, USA], and Amlexanox [Cat no: 14181; Lot no: 0584593-23; CAYMAN CHEMICAL, USA] from CAYMAN [CAYMAN CHEMICAL, Ann Arbor, MI, USA]. Lung cancer cell lines A549 and H1299 were incubated in the aforementioned inhibitors in various concentrations for three days.

In addition, experiments dealing with the exposure of cancer cells to IFNβ were conducted under a concentration of 25 ng/mL of IFNβ [ab71475; Abcam, Cambridge, UK] for 72 h. A549 and H1299 cells were seeded in 6-well plates at an approximate yield of 120,000 and 100,000 cells per well.

### 2.3. AlamarBlue^®^ Assay

Cell growth was assessed using the AlamarBlue^®^ assay for the proliferation experiments. A549 and H1299 cells were seeded in an approximate yield of 500 cells per well in a 96-well plate in triplicate for each experimental condition, following standard protocols. The cells were incubated for 24 h to facilitate attachment to the plate surface. Tofacitinib [Cat no: 11598; Lot no: 0608331-42; CAYMAN CHEMICAL, USA] was dissolved in DMSO at a concentration of 10 mM and was tested at concentrations of 1,2, and 4 μM. Ruxolitinib [Cat no: 11609; Lot no: 0568069-83; CAYMAN CHEMICAL, USA] was dissolved in absolute ethanol at a concentration of 16.32 mM, and was tested at 10, 25, and 75 nM. Finally, Amlexanox [Cat no: 14181; Lot no: 0584593-23; CAYMAN CHEMICAL, USA] was dissolved in DMSO at a concentration of 33.5 mM, and was tested at 10, 25, and 75 μM. The inhibitors were administered on Day 1 throughout the proliferation monitoring. Measurements were taken at Day 1 prior to the addition of the inhibitors, and three days after, utilizing a FLUOstar Omega microplate reader (BMG Labtech GmbH, Ortenberg, Germany).

### 2.4. Stable Transfection—shLC3A Cell Lines

H1299 cells were stably transfected with shLC3A plasmid vectors obtained from GenePharma [GenePharma; Shanghai, China]. The plasmid vectors also contained a gene conferring resistance to the antibiotic Geneticin, along with a gene that promotes the expression of the fluorescent mCherry protein. The methodology has been previously described by our research group [24]. Stably transfected cells were sorted via BD FACSAriaTM III system, in order to enhance the number of positive cells that progressively gathered within the cell culture.

### 2.5. Flow Cytometry

Flow cytometry was used to assess the membrane expression levels of HLA-class I protein in lung cancer cell lines. After exposing the lung cancer cells to the conditions previously described, the cells were collected through trypsinization, centrifuged, and washed twice with ice-cold PBS 1x. [Biosera, Cholet, France, Lot no: 017BS454]. Subsequently, incubation with an HLA-class-I ABC antibody [ab33257; PE Anti-HLA-Class-I antibody; Abcam, UK] was performed for 30 min, in the dark at room temperature, and then cells were washed twice with ice-cold PBS 1× [Biosera, Cholet, France, Lot no: 017BS454]. Sample analysis was carried out with the BD FACSAriaTM III system. Data interpretation was performed using FlowJo V10 software, with the gating strategy determined based on the unstained control for each cell line (Supplementary Materials, Figure S1). All the experiments were conducted in triplicate, and the stop recording point for each sample was set at 30,000 events in the gates of interest. Singlets were identified through FSC-H/FSC A plots, and the cell population was subsequently gated in an FSC-H/SSC-H plot. Compensation for each fluorochrome was performed before analysis, with the laser voltages optimized based on unstained or multi-stained samples.

### 2.6. Western Blot Analysis

Applying Western blot analysis, we further examined the expression pattern of HLA-class-I, alongside key autophagy-related proteins such as p62, LAMP2A, and LC3A, in A549 and H1299 lung cancer cell lines post irradiation.

The lung cancer cell lines H1299 and A549 were seeded in a T-75 culture flask to reach 50% confluency one day before treatment with ionizing radiation. Cells were exposed to ionizing radiation (IR) either as a single 8 Gy dose or through a fractionated protocol delivering 8 Gy daily over three consecutive days. Following irradiation, cells were permitted to recover for 72 h before lysates were collected, as described below for the subsequent Western blot analysis. Whole cell lysates were prepared using RIPA lysis buffer (cat# SLCD5849, Sigma-Aldrich, St. Louis, MO, USA) supplemented with protease and phosphatase inhibitors (cat# 20-201/cat# 524629, EMD Millipore Corporation, Burlington, MA, USA). The lysates were prepared on ice for 15 min with frequent agitation, followed by cell scraping and homogenization using a 20-gauge syringe needle, repeated 10 times until a uniform lysate was obtained. Protein concentrations were determined using the DC™ protein assay kit (BioRad, Hercules, CA, USA).

Equal amounts of protein (25 μg) were separated by discontinuous SDS-PAGE (9–12.5%) and transferred onto PVDF membranes (0.2 µm or 0.45 µm pore size; Merck-Millipore, Burlington, MA, USA) according to the molecular weight of the target proteins, at 50 V for 90 min at 4 °C. The membranes were then blocked with 5% non-fat dried milk in TBS-Tween for 1 h at room temperature (RT), followed by overnight incubation at 4 °C with the appropriate primary antibody. The protein expression levels of HLA class I (ABC) molecule were detected using the specific mouse monoclonal primary antibody (1:1000, ab70328, Abcam, Cambridge, UK). The primary rabbit polyclonal antibody previously validated to detect specifically LC3A (1:1000, AP1805A, Abcepta, San Diego, CA, USA), was used to detect the proform of LC3A, the LC3A-I and LC3A-II forms of the protein [19].

We further used the primary rabbit monoclonal to p62 (1:1000, ab109012, Abcam, UK), and primary polyclonal rabbit to LAMP2a (1:500, ab18528, Abcam, UK). Antibodies were applied overnight at 4 °C. After primary antibody incubation, membranes were treated with either goat anti-rabbit HRP-conjugated secondary antibody (#7074P2, Cell Signaling Technology, Danvers, MA, USA) or goat anti-mouse HRP-conjugated secondary antibody (#7076P2, Cell Signaling Technology, Danvers, MA, USA) at a 1:2000 dilution for 1 h at RT. To ensure equal protein loading, membranes were stripped using RestorePlus Western blot Stripping Buffer (#46430, Thermo Scientific, Waltham, MA, USA) and re-probed with mouse monoclonal antibody against beta-actin at a 1:5000 dilution for 1 h at RT (NB 600–501, Novus Biologicals, Centennial, CO, USA).

Protein bands were visualized using ECL Prime Luminol enhancer solution (29018903, Marlborough, MA, USA) and ECL Prime Peroxide solution (29018904, Marlborough, MA, USA) at 1:1 ratio. Immunoblot images were developed using the Chemidoc^®^ MP imaging system (BioRad, USA) and band intensity was analyzed using the ImageLab software v6.1 (BioRad, USA).

### 2.7. Co-Culturing Experiments

For the indirect co-culturing experiments, the A549 cells were seeded at an approximate yield of 10,000 cells per well in transwell inserts [SPLInsert™ Hanging, 6 Inserts/0.4 μm pores, transparent polyethylene-terephthalate (PET) membrane, Growth Area 4.52 cm^2^; SPL Life Sciences, Pochon, Kyonggi-do, Republic of Korea]. After 24 h, cells were subjected to 8 Gy of irradiation for three days. Then the inserts were placed on A549 cells seeded on 6-well plated in an approximate yield of 120,000 cells per well. The duration of the indirect co-culture of the A549 cells adherent in the lower compartment of the plate and the irradiated cells in the transwell inserts was 72 h, prior to the analysis via Flow Cytometry.

### 2.8. Real-Time PCR (RT-PCR)

For the RNA isolation protocol, the NucleoSpin^®^ RNA Plus [Lot. No: 1807/002; REF: 740,984.50; Macherey–Nagel GmbH Co. & KG, Düren, Germany] kit was utilized for RNA isolation, adhering to the instructions provided by the kit. Total RNA quantification was performed using the NanoDrop 2000 C (Thermo Fisher Scientific, Waltham, MA, USA), and total RNA samples (500 ng) were used for cDNA synthesis by the Primer-Script RT Reagent Kit (#RR037A; Takara, Shiga, Japan). The expression levels of specific genes were assessed through real-time quantitative PCR using the SensiMix SYBR No-ROX Kit, along with the [KAPA SYBR FAST qPCR kit; # KK4611; KAPA Biosystems Inc., Roche, Basel, Switzerland], which was optimized for the LightCycler^®^ 480 [KK4611, KapaBiosystems, Woburn, MA, USA]. For each sample, three measurements of the transcriptional activity of the gene of interest were recorded, with normalization based on a gene exhibiting consistent transcriptional activity across all samples. The relative changes in transcriptional levels of the genes of interest were calculated using the comparative Ct method (2^−ΔΔCt^), assuming that the efficiencies between the target genes and the reference gene are approximately equal and close to 100%. The primers for the genes of interest human MX1 gene (primer sequence 5′ → 3′ forward/reverse: CCATATTTCAGGGATCTGC/GCTCCTCTGTTATTCTCTG), human MX2 gene (primer sequence 5′ → 3′ forward/reverse: AGTATCGAGGCAAGGAGC/ACGTTAATGAAAGCTTGCTG), human UBE2L6v2 gene (primer sequence 5′ → 3′ forward/reverse: CGTCTCCGCACAAAGACC/GCAGGTTGAAGGCTTTCAGG), human IFI44 gene (primer sequence 5′ → 3′ forward/reverse: ACAGATGTTGTAATCAAGGGCC/GGTGTACATAGTCCTAGTTTCC), human IFI6v2 gene (primer sequence 5′ → 3′ forward/reverse: CAGGTGAGAATGCGGGTAAG/ATCGCAGACCAGCTCATCAG) were designed using the Roche primer design tool (https://www.hyperdesign.com/#/, accessed on 11 December 2025). The human HPRT housekeeping gene (primer sequence 5′ → 3′ forward/reverse: TGACCTTGATTTATTTTGCATACC/CGAGCAAGACGTTCAGTCCT) was used for normalization.

### 2.9. Statistical Analysis

Statistical analysis and graphical representation were conducted using the GraphPad Prism Version 8.0 software package (GraphPad Software Inc., La Jolla, CA, USA). The Wilcoxon matched pair signed rank test or the unpaired t-test with Welch’s correction was used for comparisons, as appropriate, and a *p*-value of less than 0.05 was established as the threshold for statistical significance.

## 3. Results

### 3.1. Radiation and HLA-Class-I Expression

**Figure 1:**
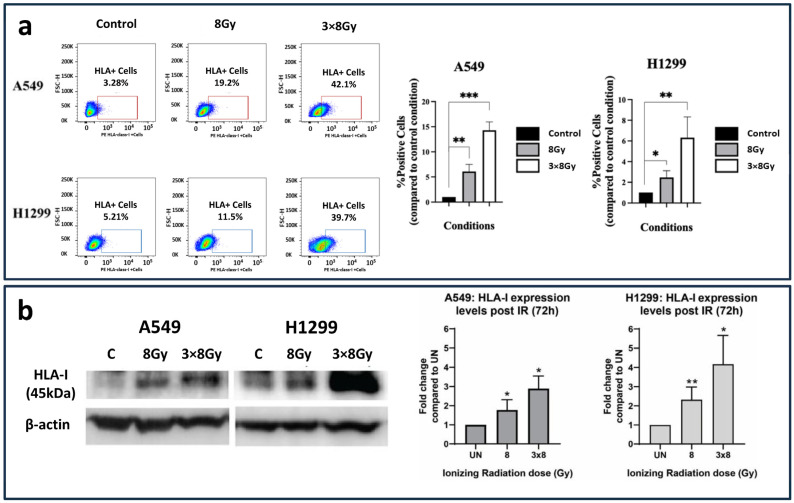
HLA-class-I expression patterns following irradiation with 8 Gy and three daily fractions of 8 Gy. (**a**) Flow cytometry images and analysis (bars show mean values with standard deviation) (fold increase compared to control ± SD) (* *p* < 0.05, ** *p* < 0.01, *** *p* < 0.001). (**b**) Western blot images and analysis (fold increase compared to control ± SD) of HLA-class-I protein expression in A549 and H1299 lung cancer cell lines exposed to a single 8 Gy dose or a fractionated ionizing radiation (IR) regimen consisting of three doses of 8 Gy. The HLA-class-I protein expression was calculated 72 h post-treatment with IR and detected as a 45 kDa protein (* *p* < 0.05, ** *p* < 0.01).

To examine the impact of RT on HLA-class-I expression, we utilized flow cytometry and Western blot analysis. Three days after irradiation, the inductive effect on HLA-class-I protein expression on the surface of the cell membranes was examined on both A549 and H1299 cell lines via flow cytometry. The percentage of A549 cancer cells with HLA-class-I expression in the gate of interest was increased from 3.28% to 19.2% (6.09-fold increase; *p* < 0.01) and 42.1% (14.29-fold increase; *p* < 0.001) post-treatment with a single dose of 8 Gy and three fractions of 8 Gy, respectively. Similarly, as far as the H1299 cell line is concerned, the HLA-class-I expression was increased from 5.21% to 11.5% (2.47-fold increase; *p* < 0.05) and 39.7% (6.31-fold increase; *p* < 0.05) post-treatment with 8 Gy and 3 × 8 Gy, respectively, as shown in Figure 1a.

Western blot analysis (Figure 1b) revealed a dose-dependent upregulation of HLA-class-I protein expression in both A549 and H1299 lung cancer cell lines following ionizing radiation (IR). In A549 cells, a single IR dose at 8 Gy resulted in a 2-fold increase in HLA-class-I expression at 72 h post-treatment, whereas the fractionated regimen of 3 × 8 Gy induced a 3-fold increase. In H1299 lung cancer cells, the single dose of IR at 8 Gy led to a 2-fold increase in HLA-class-I expression, while the fractionated regimen of three consecutive 8 Gy doses resulted in a 4.5-fold increase, as measured 72 h post-irradiation.

### 3.2. IFNβ Induces HLA-Class-I Expression

**Figure 2:**
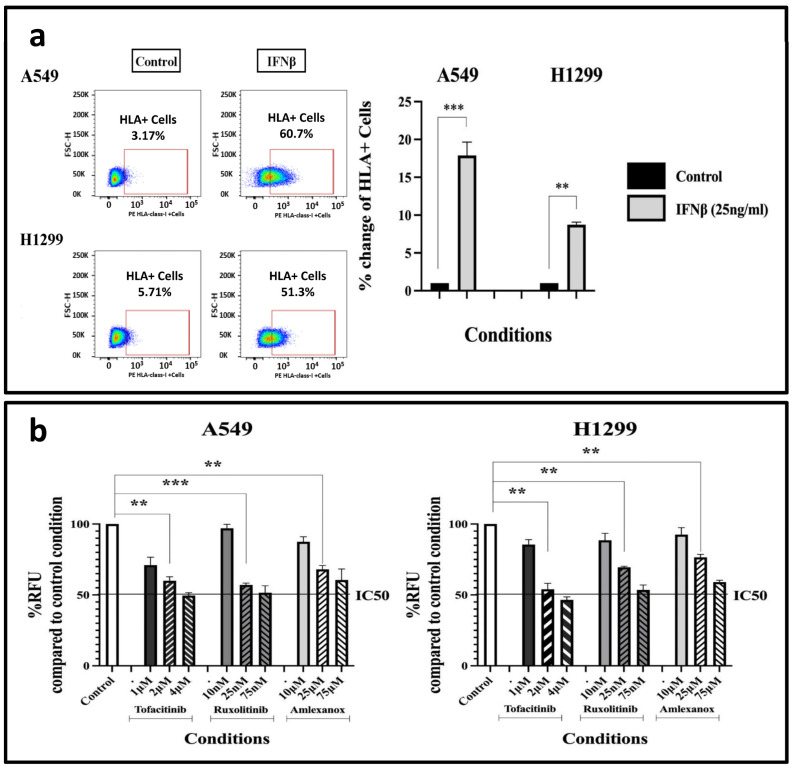
(**a**) Flow-cytometry HLA-class-I expression patterns following a 3-day (72 h) exposure of A549 and H1299 cell lines to 25 ng/mL IFNβ (fold increase compared to control ± SD) (** *p* < 0.01, *** *p* < 0.001). (**b**) AlamarBlue^®^ assay to assess the impact of a 3-day (72 h) incubation with different concentrations of IFN-type-I-response inhibitors on A549 and H1299 cell proliferation (fold increase compared to control ± SD) (** *p* < 0.01, *** *p* < 0.001). Abbreviations: RFU = Relative Fluorescence Units, IC50 = Half maximal inhibitory concentration.

Although the inducing effect of type I IFNs on HLA class I expression is well established, we performed confirmatory experiments to demonstrate that the A549 and H1299 cancer cell lines are indeed responsive to IFNβ. Treatment of the cells for 72 h with 25 ng/mL IFNβ resulted in a rise in the proportion of cancer cells expressing HLA-class-I protein compared to the control condition, with the A549 cell line showing a 17.87-fold increase (*p* < 0.001) and the H1299 cell line demonstrating an 8.74-fold increase (*p* < 0.01) in the expression pattern; see Figure 2a.

### 3.3. The Effect of IFN-Type-I-Response Inhibitors on Cell Survival and Proliferation

To select the appropriate concentration of IFN-type-I-response inhibitors for further experiments, a cell toxicity assay was conducted to prevent choosing a highly toxic drug dose.

We studied the effect of various concentrations of Tofacitinib (Janus kinase (JAK) inhibitor targeting the JAK1 enzyme), Ruxolitinib (Janus-associated Kinase 1 and 2 inhibitor) and Amlexanox (TBK1 and IKKε inhibitor) on cell survival and proliferation, via the AlamarBlue^®^ assay. Following multiple testing experiments, A549 and H1299 cells were exposed to chosen concentrations of 1 to 4 μΜ of Tofacitinib, 10–75 nM of Ruxolitinib, and 10–75 μΜ of Amlexanox, for three days (Figure 2b). Based on the results, we selected specific drug concentrations that produce relatively low cytotoxicity (lower than the IC50), allowing experiments involving HLAs to be performed in combination with drug concentrations of minimal toxicity. Thus, 2 μΜ of Tofacitinib resulted in a 0.6-fold decrease in cell proliferation (*p* < 0.01) in the A549 cell line, and a 0.54-fold decrease in the H1229 one (*p* < 0.01), compared to the control condition. Similarly, 25 nM of Ruxolitinib resulted in a 0.57- (*p* < 0.001) and 0.695-fold (*p* < 0.001) decrease; 25 μΜ of Amlexanox resulted in a 0.68- (*p* < 0.01) and 0.765-fold (*p* < 0.01) decrease on A549 and H1299 cell populations, respectively. These concentrations were subsequently used for HLA-class-I experiments.

### 3.4. Effect of IFN-Type-I-Response Inhibitors and RT on IFNβ mRNA Levels

Although the drugs examined in this study have been validated as IFN-type-I pathway inhibitors and their impacts on IFNβ and downstream events are predictable, the effects of combining them with RT remain unknown. Additionally, not all cells increase IFNβ secretion, as downstream IFN responses can be activated through pathways independent of cGAS-STING [30]. Therefore, using RT-PCR, we assessed the effect of 2 μΜ of Tofacitinib, 25 nM of Ruxolitinib, and 25 μΜ of Amlexanox on A549 and H1299 cells.

**Figure 3:**
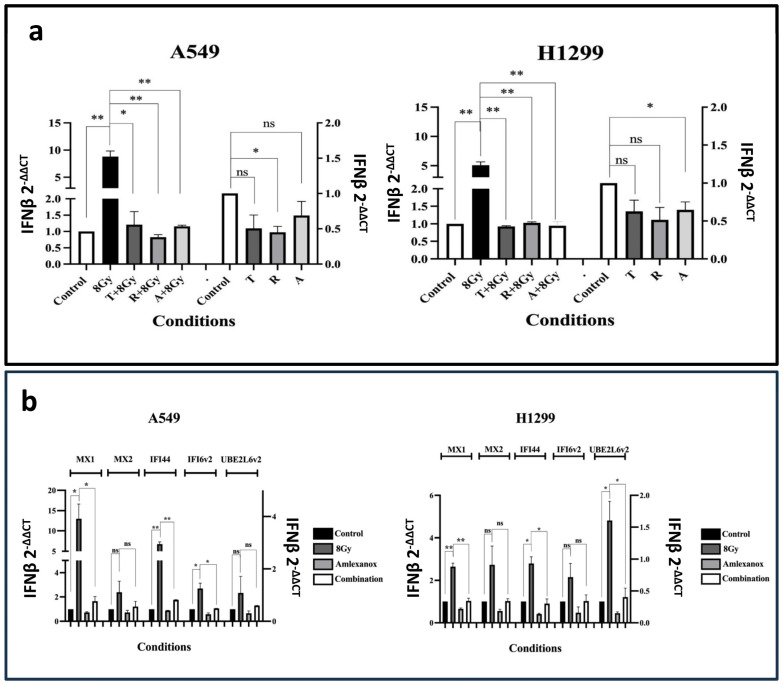
(**a**) RT-PCR results of the IFNβ mRNA expression levels after three days of exposure to 2 μΜ of Tofacitinib (T), 25 nM of Ruxolitinib (R), and 25 μΜ of Amlexanox (A) with or without 8 Gy of irradiation on A549 and H1299 cell lines (fold change ± SD) (* *p* < 0.05, ** *p* < 0.01, ns = not significant). (**b**) RT-PCR results of the mRNA expression levels of ISGs MX1, MX2, IFI44, IFI6v2, and UBE2L6v2 after 8 Gy RT, 3 days incubation (72 h) with 25 μΜ of Amlexanox or its combination with RT (combination) on A549 (**a**) and H1299 (**b**) cell lines (fold change compared ± SD) (* *p* < 0.05, ** *p* < 0.01).

Incubation of cells with the Tofacitinib and Amlexanox inhibitors alone did not result in statistically significant changes compared to the control condition; meanwhile, incubation with 25 nM of Ruxolitinib resulted in a 0.45-fold decrease in IFNβ mRNA levels (*p* < 0.05); see Figure 3a. The same pattern was recorded in the H1299 cell line experiments; see Figure 3a. Although Amlexanox was expected to have the most potent activity on IFNβ expression (see methods), Ruxolitinib, which is expected to act downstream of IFNβ expression, showed more potent activity. This may indicate additional effects on upstream molecular targets or an effect resulting from feedback loops.

We further assessed the effect of combining RT with IFN-type-I inhibitors on IFNβ gene expression; 8 Gy of radiation resulted in an intense increase in IFNβ mRNA levels (*p* < 0.01). Combining 8 Gy of irradiation with the IFN-type-I-response inhibitors completely blocked the inductive effect of RT to IFNβ (*p* < 0.05); see Figure 3a. This finding was quite unexpected; with the exception of Amlexanox, these drugs are not typically thought to inhibit IFNβ production, further indicating that they could also be used to target the upstream components of the IFN pathway.

### 3.5. Exposure to RT and Amlexanox on ISG Expression

To examine the impact of blocking IFN-type-I on RT-induced ISGs, we selected Amlexanox, a drug currently being investigated in clinical trials for various diseases, including cancer [31]. Amlexanox, as a TBK1-IKKe inhibitor, acts on the converging pathway of IFN-I cellular response, inhibiting IFN production directly.

RT with 8 Gy induced the MX1, IFI44 and IFI16v2 mRNA levels in the A549 cell line (*p* < 0.05), and the MX1, IFI44 and UBE2L6v2 in the H1299 cell line (*p* < 0.05); see Figure 3b. Co-administration with Amlexanox completely abrogated the inductive effect of RT (*p* < 0.05). A slight reduction in mRNA expression compared to the control was noted after incubation of non-irradiated cells with Amlexanox.

### 3.6. Effect of IFN-Type-I-Response Inhibitors and RT on HLA-Class-I Expression

We used flow cytometry to investigate whether IFN-type I pathway inhibitors suppress the expression of HLA class I molecules in lung cancer cell lines. Additionally, since the RT-induced IFN-type I response is ultimately involved in increasing HLA-class I expression in cancer cells, we tested whether IFN-type I inhibitors could block this effect of RT.

**Figure 4:**
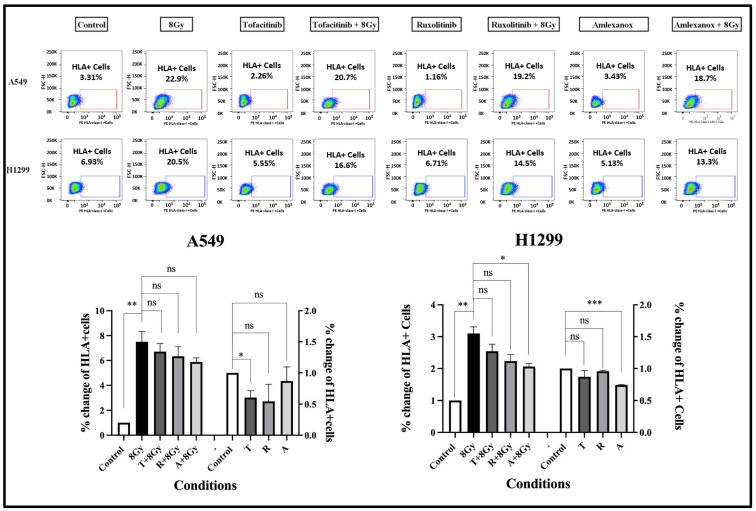
Flow cytometry images and analysis of the expression levels of HLA-class-I protein after three days (72 h) of exposure to 2 μΜ of Tofacitinib, 25 nM of Ruxolitinib, and 25 μΜ of Amlexanox with or without 8 Gy of irradiation, in A549 and H1299 cell lines (fold change ± SD) (* *p* < 0.05, ** *p* < 0.01, *** *p* < 0.001, ns = not significant).

The effect of 8 Gy RT, with or without IFN-type-I-response inhibitors, on HLA-class-I expression was assessed after three-day incubation, using flow cytometry. In the A549 cell line, 8 Gy of RT resulted, as expected, in an intense increase in HLA-class-I+ cells from 3.31% to 22.9% (7.5-fold increase; *p* < 0.01); see Figure 4. The three drugs had a minor blocking effect on HLA induction in irradiated cells; see Figure 4. Incubation of unirradiated cells with the inhibitors slightly reduced the expression of HLA-class-I molecules. Analogous results were obtained in experiments with the H1299 cell line; see Figure 4.

### 3.7. Co-Culturing of A549 Cells with Irradiated Cells

A question was raised about whether HLA upregulation in irradiated cancer cells results from an intracellular pathway or from bystander effects mediated by cytokine secretion that could influence non-irradiated cells. To address this question, we evaluated the changes in HLA-class-I expression in A549 cells (which showed higher IFNβ production after RT) after exposing them to indirect co-culture (shared culture medium) with previously irradiated A549 cells (three fractions of 8 Gy), using flow cytometry. An increase in the percentage of cells expressing HLA-class-I antigen was noted (1.61-fold increase, compared to control untreated cells; *p* < 0.05); see the Supplementary Materials, Figure S2.

### 3.8. Irradiation and Autophagy Proteins

To examine the potential blockage of autophagy flux in A549 and H1299 lung cancer cell lines after exposure to a high RT dose of 8 Gy, we conducted Western blot analysis of key autophagy-related proteins LC3A, p62, and LAMP2A (their biology is detailed in the introduction). In a previous study, exposure to lower doses of 4 Gy elicited different responses among cell lines [13]. Specifically, A549 cells responded by blocking autophagy flux and lacking transcriptional activation of auto-lysosomal genes, whereas the more radio-resistant H1299 cell line exhibited an increase in autophagy flux. Our previous experience suggests a dose-dependent autophagic response along with cell-inherent responsiveness, so repeating experiments with 8 Gy was necessary.

**Figure 5:**
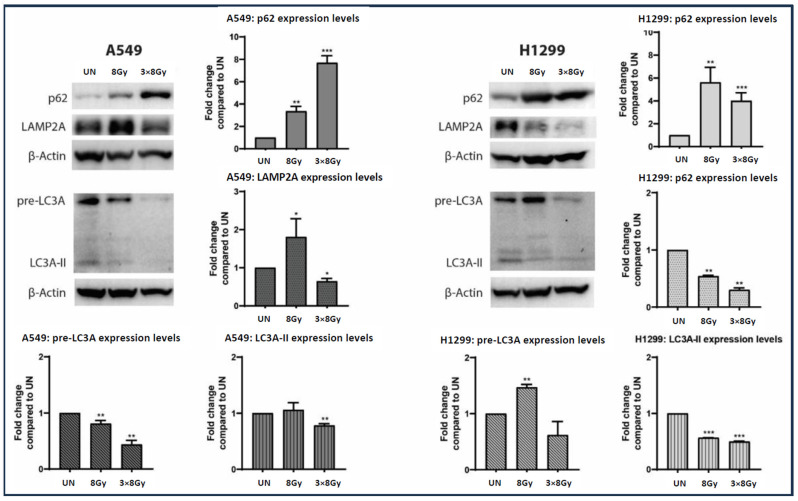
Western blot images and analysis (fold increase compared to control ± SD) of p62, LAMP2A, LC3A protein expression in A549 and H1299 lung cancer cell lines exposed to a single 8 Gy dose or a fractionated ionizing radiation (IR) regimen consisting of three doses of 8 Gy. The protein expression levels of each autophagy-related marker were calculated 72 h post treatment with IR (* *p* < 0.05, ** *p* < 0.01, *** *p* < 0.001). p62 is detected as a 62 kDa protein, LAMP2a as a 110 kDa protein, pre-LC3A as a 26 kDa protein, and LC3A-II as a 16 kDa protein.

Western blot analysis (Figure 5) showed that p62 protein expression levels were increased in both A549 and H1299 lung cancer cell lines following ionizing radiation (IR). In A549 cells, a single IR dose at 8 Gy resulted in a 4-fold increase in p62 expression at 72 h post-treatment, whereas the fractionated regimen of 3 × 8 Gy induced an 8-fold increase. In H1299 lung cancer cells, the single dose of IR at 8 Gy led to a 6-fold increase in p62 expression, while the fractionated regimen of three consecutive 8 Gy doses resulted in a 4-fold increase, as measured 72 h post-irradiation.

In A549 cells, LAMP2A protein levels were upregulated by 1.8-fold at 8 Gy of irradiation, while treatment with 3 × 8 Gy resulted in a 0.5-fold downregulation. In H1299 cells, LAMP2A protein levels exhibited a dose-dependent decrease of 0.5-fold at 8 Gy of irradiation and 0.75-fold at 3 × 8 Gy regimen.

Moreover, in A549 cell lines exposed to 8 Gy of irradiation, pre-LC3A demonstrated a 0.25-fold decrease, accompanied by no significant alteration in LC3A-II protein levels, while treatment with 3 × 8 Gy of irradiation resulted in a 0.5-fold and 0.25-fold decrease for pre-LC3A and LC3A-II, respectively. In H1299 cells, treatment with 8 Gy of irradiation resulted in a 1.5-fold elevation of pre-LC3A with a parallel 0.5-fold decrease in LC3A-II protein. Exposure of H1299 cells to a 3 × 8 Gy regimen showed a decreasing trend of pre-LC3A, accompanied by a significant 0.5-fold reduction in LC3A-II protein.

### 3.9. Experiments with Autophagy-Impaired Cell Lines and Irradiation

To study the basal levels of HLA-class-I expression in autophagy-compromised lung cancer cells and the comparative effect of RT on these cells, stably transfected H1299 shLC3A cells and ATG7-deficient H1650 cells were studied. The levels of HLA-class-I in unirradiated and irradiated cells, following a single dose of 8 Gy, were assessed using flow cytometry.

**Figure 6:**
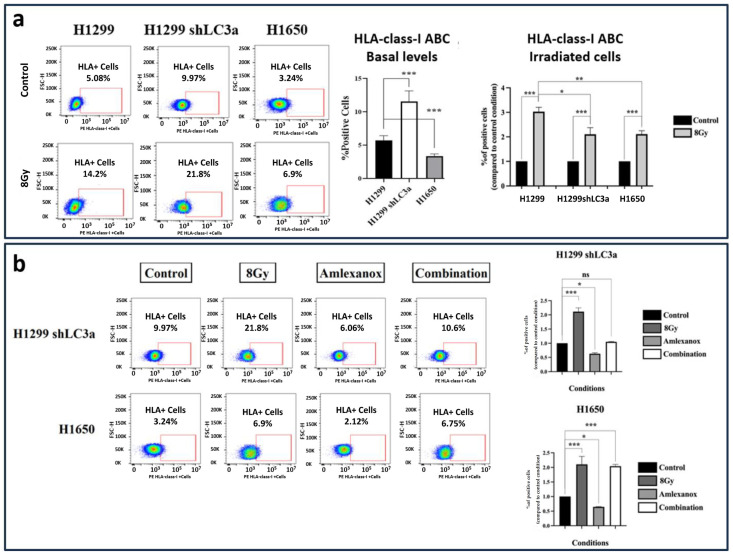
Flow Cytometry images and analysis of HLA-class-I expressing cells in the H1299 wild type, the stably transfected H1299-shLC3A, and ATG7 deficient H1650 cell line: (**a**) three days (72 h) after exposure to a single dose of 8 Gy and (**b**) after exposure to Amlexanox and 8 Gy RT combinations (* *p* < 0.05, ** *p* < 0.01, *** *p* < 0.001, ns = not significant).

H1299 shLC3A cells exhibited higher basal HLA-class-I levels than parental H1299 cells (2.02-fold higher; *p* < 0.001). The basal levels of ATG7-deficient H1650 cells were 0.59-fold lower than those of H1299 cells (*p* < 0.001); see Figure 6a.

The percentage of H1299 wild-type cells expressing HLA-class-I in the gate of interest increased from 5.08% to 14.2% after 8 Gy of irradiation (2.8-fold increase; *p* < 0.05). Exposure of shLC3A cells to 8 Gy of irradiation increased the percentage of HLA-class-I positive cells from 9.97% to 21.8% (2.1-fold increase; *p* < 0.001), despite the already increased basal levels compared to the parental cells. This inductive effect of radiation (increase above basal levels), however, was significantly lower than that detected in the wild-type cells (*p* < 0.05); see Figure 6a. Furthermore, exposure of the shLC3A cells to 25 μΜ of Amlexanox appeared to reduce the percentage of cells expressing HLA-class-I to 6.06% (1.59-fold decrease; *p* < 0.05). The inductive effect of radiation was abrogated in combination with 25 μM of Amlexanox; see Figure 6b.

Similarly, a single dose of 8 Gy of RT increased the percentage of positive HLA-class-I cells in the H1650 cell line compared to the untreated cells (from 3.24% to 6.9%; 2.1-fold increase; *p* < 0.001); see Figure 6a. Again, exposure to 25 μΜ of Amlexanox appeared to reduce the percentage HLA-class-I+ cells to 2.12% (1.54-fold decrease; *p* < 0.05). In contrast to shLC3A cells, the HLA induction in irradiated H1650 cells was not blocked by the 25 μΜ of Amlexanox, as the percentage of cells expressing HLA-class-I was 6.75% (2-fold increase, compared to untreated cells; *p* < 0.001), and non-statistically significant changes were detected compared to the 8 Gy of RT alone; see Figure 6b.

## 4. Discussion

After 120 years of experimental and clinical research in cancer immunology, the discovery of immune checkpoint pathways and the development of monoclonal antibodies to interfere with complex immune system–tumor interactions have ushered in a new era of cancer immunotherapy. Anti-CTLA4 and anti-PD-L1/PD-1 therapeutic antibodies have revolutionized the field of clinical oncology, enabling physicians to advance therapy beyond traditional chemotherapy and RT, thereby increasing curability and prolonging the survival of patients with previously incurable cancers. The astonishing success rates in certain tumors, such as those with Mismatch Repair Deficiency (dMMR), encourage further research and strengthen the hypothesis that cancer can be cured by manipulating the body’s immune machinery [32].

Despite the impressive responses with anti-PD-1/PD-L1 IO in a subgroup of patients with advanced NSCLC, the median progression-free survival does not exceed 9 months [33]. Several explanations for the failure of tumors to respond or the development of resistance to IO have been postulated [34]. The prevalence of additional, less studied immune-checkpoint pathways may define inherent resistance to the available anti-PD-L1/PD-1 and anti-CTLA4 monoclonal antibodies. An intra-tumoral microenvironment unfavorable for T-cell proliferation and activation, established for various reasons, including hypoxia and acidity, or enrichment by immunosuppressive metabolites such as adenosine and kynurenine, accounts for the inherent resistance. Acquired resistance appears to be a complex phenomenon, encompassing post-treatment changes in tumor biology and the tumor microenvironment, as well as alterations in systemic immune response patterns.

Defects in antigen presentation may contribute to both inherent and acquired immune resistance. In a previous study, we found that lung cancer cells exhibit a complete or extensive loss of HLA-class-I antigen expression in 64% of NSCLC patients [10]. Experimental studies suggest that the development of resistance to anti-PD-1 IO does not occur through changes in PD-L1 expression, but rather through the loss of MHC-class-I antigen expression [26]. Moreover, tumor irradiation restored anti-PD-1 therapeutic activity by upregulating the IFN-type I response and re-expression of MHCs [26]. A similar pattern of restored HLA-class-I expression in response to RT has also been reported in a clinico-pathological study we conducted on rectal cancer [11]. Since HLA-loss is common in most NSCLC cases [12] and may be a key reason for IO failure, radiation therapy (RT) as a way to restore HLA-mediated tumor antigen presentation becomes a promising therapeutic strategy. The improvement in the survival rates of stage III NSCLC patients treated with post-RT anti-PD-1 therapy [4], or even the significant enhancement of abscopal effects and survival of patients with metastatic NSCLC treated with anti-PD-1 therapy in combination with irradiation of metastatic disease [33], may be attributed, at least in part, to radiation-mediated HLA upregulation.

Considering the significant interaction between RT and cancer cell recognition by the immune system, we examined potential mechanisms involved in HLA upregulation, aiming to identify targets for therapeutic intervention to improve ICI efficacy. In the first step, we confirmed that irradiation of NSCLC cell lines with a single fraction of 8 Gy increased HLA-expressing cancer cells 6-fold, which reached 14-fold enhancement after three fractions of 8 Gy. Indirect co-culturing of unirradiated and irradiated cancer cells showed a significant, although of lesser magnitude, induction of HLAs in non-irradiated cells, supporting a by-stander effect. This suggests that chemical factors released by irradiated lung cancer cells, e.g., IFNβ released by cancer cells [35,36] may induce HLA in adjacent non-irradiated cancer cells. However, it remains unclear whether this ultra-hypo-fractionated RT scheme of 8 Gy is more efficient than the conventional 2 Gy per fraction schedule applied in the current clinical practice for the treatment of lung cancer in inducing HLAs. Nevertheless, in a recent study from our group, treatment of locally recurrent NSCLC with three fractions of 8 Gy was feasible and resulted in high complete response rates [24].

Alongside HLA-class-I upregulation, we found that 8 Gy of RT caused a 5–8-fold increase in IFNβ mRNA levels and significantly boosted the mRNA expression of several ISGs. This result was expected but was confirmed in our two cell lines as validation for further experiments. The induction of the IFN-type-I response by RT was demonstrated in a seminal study by Burnette et al. in 2011 [35]. Vanpouille-Box et al. have more recently shown that large RT fractions in the range of 8 Gy produce a robust induction of the IFN-type I response [36]. Since type I IFNs are well known to induce HLA-class I expression [37], as also documented here for our two lung cancer cell lines, we hypothesized that RT-mediated induction of type I IFNs could contribute to the upregulation of HLA. To test this hypothesis, we conducted experiments using three inhibitors of the type I IFN response that target JAK and TBK1 pathways.

Indeed, all three drugs could inhibit the induction of the IFN-type I response and ISG transcription in irradiated cancer cells, making it feasible to test whether this repressive effect could prevent HLA induction by RT. However, this hypothesis was found to be invalid. Blocking the IFN-type-I response with specific inhibitors, while completely suppressing IFNβ and ISG expression, was not enough to reduce HLA expression after RT. Thus, it was concluded that while the IFN-type-I response contributes to HLA upregulation caused by RT, other pathways are also involved.

Considering recent evidence that autophagy blockage increases HLA-class-I levels [12,15], along with previous observations that RT may inhibit autophagy [15,25], we hypothesize that the autophagy impairment caused by a large fraction of 8 Gy could be an additional pathway linked to HLA expression. Western blot experiments confirmed the radiation-induced blockage of autophagy by analyzing changes in key proteins involved in autophagy flux. The dense buildup of p62 in both cell lines suggests an impairment of autophagic degradation, since p62, which transports proteins to autophagosomes, is degraded after autophagosome–lysosome fusion [23]. Nevertheless, transcriptional upregulation of p62 could also lead to overexpression of the p62 protein. However, our previous studies suggest that p62 mRNA levels increase sharply around 7 days after irradiation, coinciding with a recovering activation of autophagic flux following an initial blockage that occurs 2–4 days after RT [15,25]. In addition, LC3A protein, both its pre-LC3A cytoplasmic form and LC3A-II autophagosome membrane form, was also reduced alongside the lysosomal membrane protein LAMP2A, indicating a suppression of these key autophagy proteins.

We further tested the above hypothesis in a cell line with LC3A impaired autophagy produced by an shRNA-mediated stable knockout technique. This cell line exhibited higher steady-state HLA-class I levels than the parental line, further suggesting a role of LC3A in HLA-class-I expression. Treatment of the shLC3A cell line with 8 Gy resulted in a significant increase in HLA-class-I expression; however, this effect was of significantly lesser magnitude compared to control cells with intact autophagy, eventually due to the already high HLA basal levels. It is suggested that the autophagy-irrelevant pathway, triggered by RT-induced IFN-type-I response, enhances HLA-class-I expression in this autophagy-deficient cell line. Indeed, IFN-type-I-response inhibitors completely abrogated HLA induction by RT. However, an ATG7-deficient cell line showed different patterns. The low basal levels of HLA-class-I may suggest that ATG7-independent autophagy may still sustain high LC3A levels. This may also explain the finding that radiation-mediated autophagy blockage enhances HLA-class-I expression, despite the addition of IFN-type-I-response inhibitors. This observational finding requires further experiments, which are beyond the scope of the current study, to reveal insights into the underlying mechanisms.

Any experimental approach for investigating the RT/autophagy interplay on the HLA-class-I expression is complex and demands far more steps than those outlined herein. Induction of type I IFN by RT could also directly influence autophagy [38]. IFNs have been demonstrated to induce autophagy in a concentration-dependent manner within 48 h of treatment. The type I IFN binds to its receptors, triggering the phosphorylation of STAT1/2 through JAK1 and TYK2. After they combine with IRF9 to form the complex transcriptional activator ISGF3, transcription of ISGs occurs. JAK1 and STAT2 are crucial for IFNα to trigger autophagy. Therefore, IFNs may enhance autophagy and inhibit HLA induction after irradiation, but this was not observed in our experiments. It is possible that the autophagy blockage caused by high doses of RT in our study inhibited autophagy, which, in turn, prevented the IFN-mediated induction of autophagy. Nevertheless, repeated daily lower RT doses that also trigger IFN-type I responses [30] may enhance autophagy, so IFN-type I inhibitors could be useful in conventional low-dose per-fraction RT. This interplay among IFNs, autophagy, and RT is definitely a complex phenomenon that depends on cell type, RT fractionation, and total dose.

## 5. Conclusions

The current study suggests that RT increases the expression of HLA-class-I molecules in lung cancer cell lines in parallel with the upregulation of the IFN-type-I response pathway. Although IFNβ strongly induces overexpression of HLA-class-I molecules, pharmaceutical abrogation of the IFN-type-I response did not block the inductive effect of radiation. Blockage of the LC3A-mediated autophagic flux is postulated to be an additional pathway that radiation exploits to induce HLA-class-I molecules. This interplay between RT, IFN-type-I response, LC3A-mediated autophagy, and HLA-class-I expression by cancer cells provides a promising target for the development of radio-vaccination strategies aiming to enhance the efficacy of radio-immunotherapy. For example, combining chloroquine with RT and ICIs could enhance the effectiveness of immune-RT, while adding RT, chloroquine, and/or IFNα to standard ICI therapy at relapse may restore the immune recognition of cancer cells and extend the disease remission already achieved by ICIs.

## Data Availability

Research data are stored in an institutional repository and will be shared upon request to the corresponding author.

## Supplementary Materials

The following supporting information can be downloaded at: https://www.mdpi.com/article/10.3390/cimb48010028/s1.
