# Supplementary figures and images for: Interferon-Type-I Response and Autophagy Independently Regulate Radiation-Induced HLA-Class-I Molecule Expression in Lung Cancer
Source: Curr Issues Mol Biol. 2025 Dec 25;48(1):28. doi: 10.3390/cimb48010028 (PMC12840286; doi:10.3390/cimb48010028)

## Supplemental Figure S1

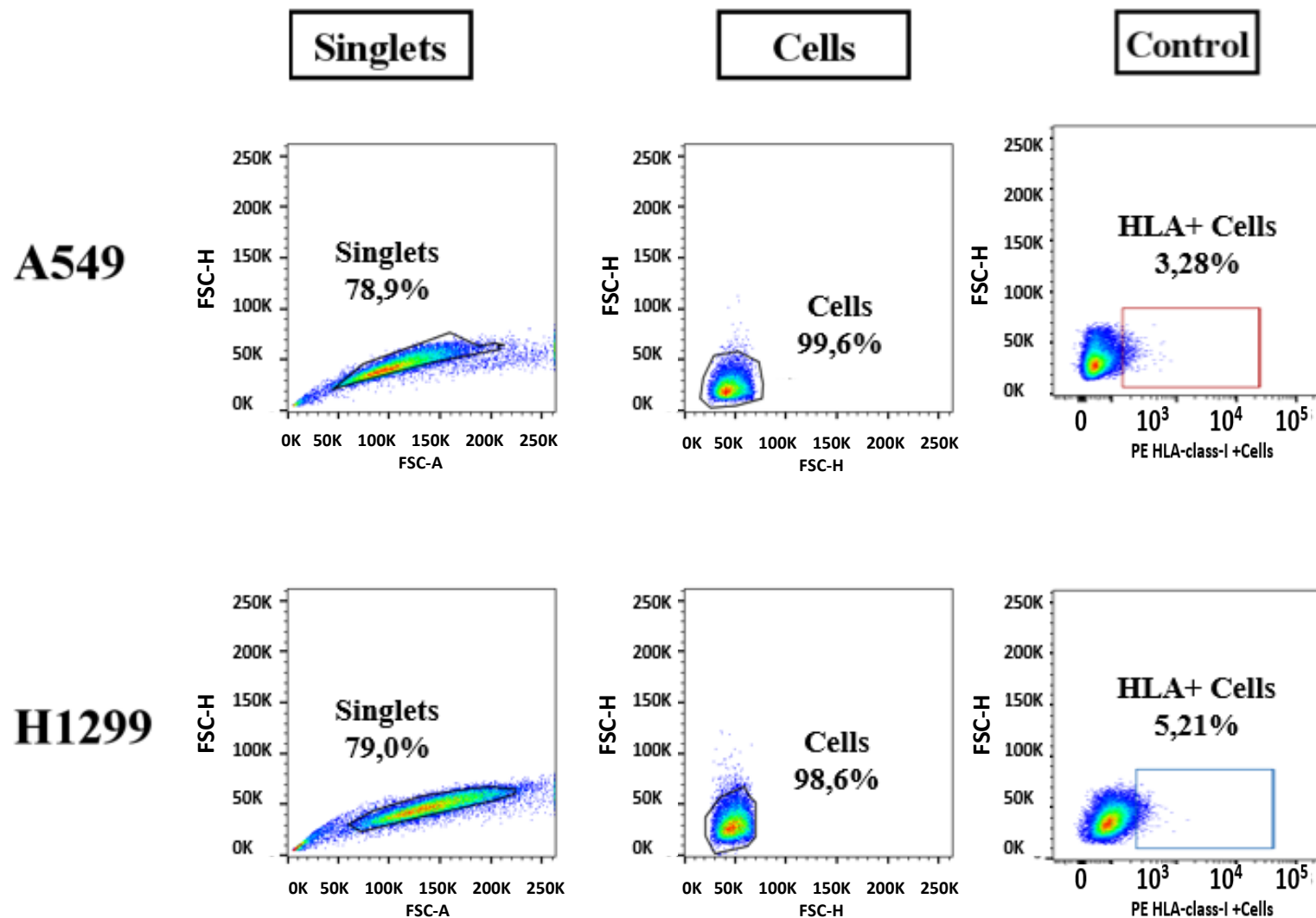

Supplement: Supplementary file 1 [file cimb-48-00028-s001.zip › Suppl Figure S1 pdf.pdf]

Supplemental Figure S2

**A549**

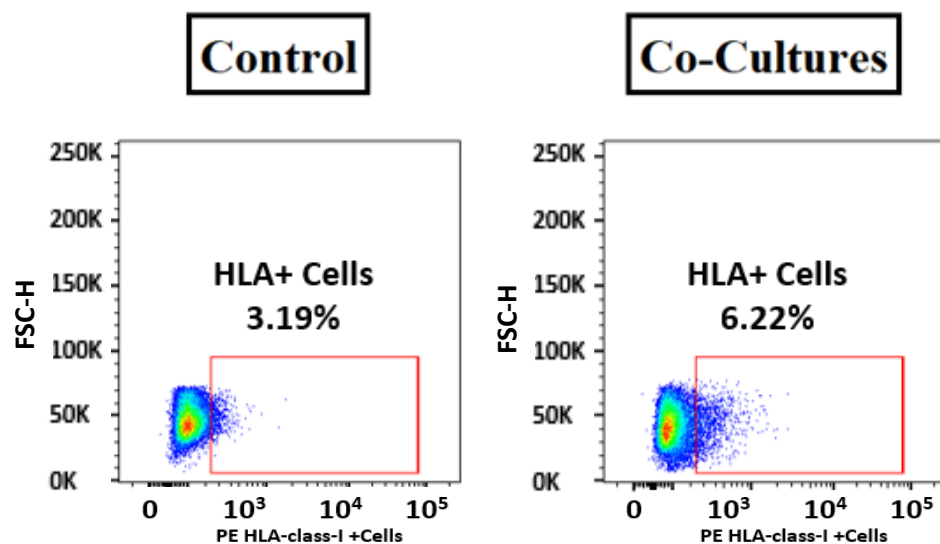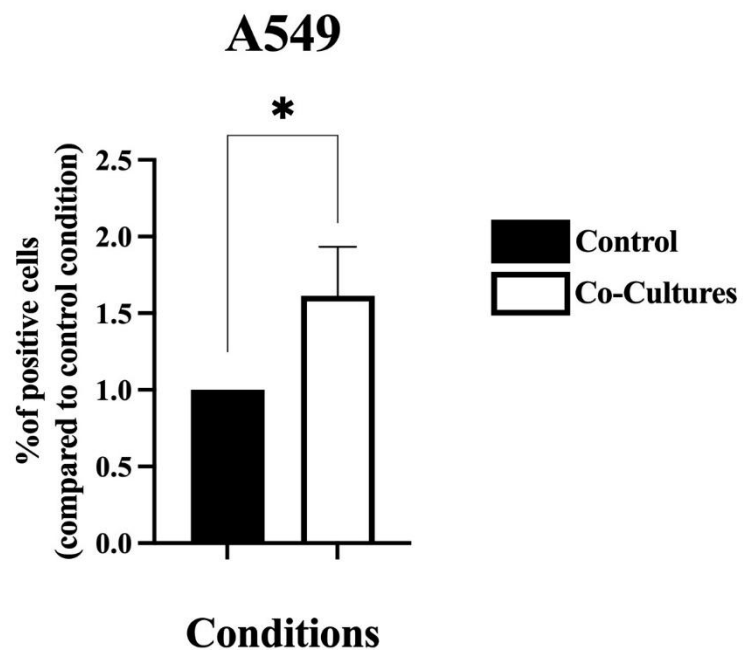

Supplement: Supplementary file 1 [file cimb-48-00028-s001.zip › Suppl Figure S2 pdf.pdf]
